# Legacy Effects on the Recovery of Soil Bacterial Communities from Extreme Temperature Perturbation

**DOI:** 10.3389/fmicb.2017.01832

**Published:** 2017-09-25

**Authors:** Stephanie D. Jurburg, Inês Nunes, Asker Brejnrod, Samuel Jacquiod, Anders Priemé, Søren J. Sørensen, Jan Dirk Van Elsas, Joana F. Salles

**Affiliations:** ^1^Microbial Ecology Group, Groningen Institute for Evolutionary Life Sciences, University of Groningen Groningen, Netherlands; ^2^Bioinformatics group, Bioveterinary Institute, Wageningen University and Research Wageningen, Netherlands; ^3^Section of Microbiology, University of Copenhagen Copenhagen, Denmark; ^4^Microbe Technology Department, Novozymes Copenhagen, Denmark

**Keywords:** secondary succession, soil bacteria, microcosm, disturbance, resilience, RNA

## Abstract

The type and frequency of disturbances experienced by soil microbiomes is expected to increase given predicted global climate change scenarios and intensified anthropogenic pressures on ecosystems. While the direct effect of multiple disturbances to soil microbes has been explored in terms of function, their effect on the recovery of microbial community composition remains unclear. Here, we used soil microcosm experiments and multiple model disturbances to explore their short-term effect on the recovery of soil microbiota after identical or novel stresses. Soil microcosms were exposed to a heat shock to create an initial effect. Upon initial community recovery (25 days after stress), they were subjected to a second stress, either a heat or a cold shock, and they were monitored for additional 25 days. To carefully verify the bacterial response to the disturbances, we monitored changes in community composition throughout the experiment using 16S rRNA gene transcript amplicon sequencing. The application of a heat shock to soils with or without the initial heat shock resulted in similar successional dynamics, but these dynamics were faster in soils with a prior heat shock. The application of a cold shock had negligible effects on previously undisturbed soils but, in combination with an initial heat shock, caused the largest shift in the community composition. Our findings show that compounded perturbation affects bacterial community recovery by altering community structure and thus, the community’s response during succession. By altering dominance patterns, disturbance legacy affects the microbiome’s ability to recover from further perturbation within the 25 days studied. Our results highlight the need to consider the soil’s disturbance history in the development of soil management practices in order to maintain the system’s resilience.

## Introduction

Ecosystems are expected to face increasing anthropogenic pressures and climatic oscillations (Millenium Ecosystem Assessment, 2005; Hartmann et al., 2013; Trenberth et al., 2014), but how these changes will affect the soil biota is poorly understood (Smith et al., 2015). For instance, despite their critical contribution to ecosystem services, the precise role of the soil microbiota in safeguarding the soil processes under increased environmental constraints is largely unknown (Nemergut et al., 2014). Particularly, we lack evidence on the influence of altered soil microbial community structures on the stability of soil functioning (McGuire and Treseder, 2010; Nemergut et al., 2014). Microbial communities, both in the field and in micro/mesocosm experiments, often exhibit long-term changes in their structure following a disturbance (Allison and Martiny, 2008; Shade et al., 2012). These altered community compositions may be ecologically relevant if interactions between populations are ruptured or if the community’s ability to resist invasion is affected, for example, as has been recently shown (Griffiths et al., 2007; van Elsas et al., 2012; Fiegna et al., 2015; Mallon et al., 2015).

In particular, the increasing frequency of transient disturbances in soil ecosystems, resulting in compounded perturbation, represents a challenge for research. Compounded perturbation is defined as an ecosystem being stressed during the recovery process from a previous disturbance event (Paine et al., 1998). It has been suggested to have a ‘multiplicative’ effect on microbial communities (Paine et al., 1998), which is defined by the combined effect of both perturbations being greater than the sum of their individual effects. We distinguish between two cases of compounded perturbation, namely (1) mixed compounded perturbation, in which the first stress event differs substantially from the second and each selects for different microbes, and (2) homogeneous compounded perturbation, in which the first and second disturbance events are of the same type, and the same microbes are selected for by both. Previous experiments have found a multiplicative effect of mixed compounded perturbations (Kuan et al., 2006; Tobor-Kaplon et al., 2006). In these experiments, soils subjected to long-term disturbance such as exposure to intensive agricultural practices or heavy metals, were exposed to an additional short-term stress, such as a temperature shock or an antibiotic (Müller et al., 2002; Tobor-Kaplon et al., 2005, 2006; Kuan et al., 2006). These compounded treatments resulted in a slower (or null) recovery of function (i.e., substrate utilization rate) relative to soils without the prior disturbance. The opposite pattern has been observed in the case of homogeneous compounded perturbation: generally, the second disturbance exerted a lesser effect on the community or its functioning than the first. For example, soils previously exposed to extreme precipitation regimes were less functionally sensitive to further moisture pulses than unexposed controls (Evans and Wallenstein, 2012). Similarly, soils underlying an oak tree exhibited shifts in bacterial community composition in response to drying-rewetting regimes, while grassland soils in the same area, which experienced more radical natural fluctuations in moisture, exhibited no change (Fierer et al., 2003). The link between community composition and function is still unclear in soil microbial communities due to the high degree of functional redundancy (Jurburg and Salles, 2015), however, a comprehensive meta-analysis has found that community composition is sensitive to disturbance (Allison and Martiny, 2008). Nevertheless, few studies focus on how perturbations alter bacterial community structure at a sufficient temporal resolution to detect community dynamics. While it is likely that disturbance shifts community composition by killing vulnerable taxa (Lennon et al., 2012), the newly open niche spaces may trigger competitive dynamics which result in sequential community shifts, similar to secondary succession (Placella et al., 2012; Jurburg et al., 2017).

**FIGURE 1:**
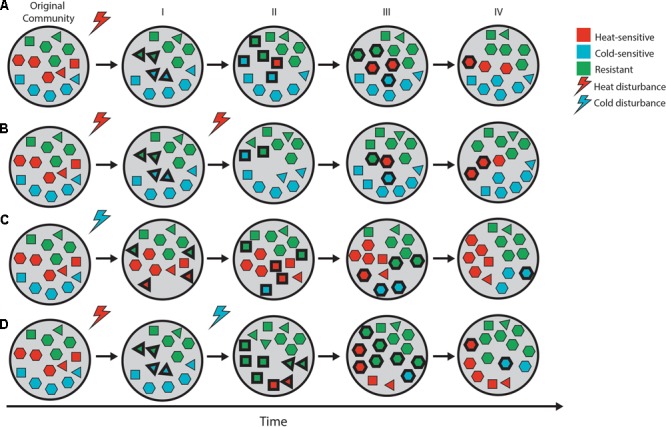
Schematic of microbial community recovery from pulse disturbance over time. Shape-color combinations represent different microbial taxa, and bold outlines highlight “new” taxa drawn from a local pool. The initial stages of community recovery are highly dependent on disturbance type (**A**—heat, **C**—cold), as sensitive individuals are removed from the system and survivors compete to consume the newly available resources (I). Over time, the sensitive taxa return to the system, compete with the survivors to increase their abundance (II, III), and may eventually reach a structure similar to that of the pre-disturbance community (IV). The effect of compounded perturbation depends on the disturbance type as well. In the case of homogeneous perturbation **(B)**, the second disturbance has little effect on the community, as the sensitive organisms have already been removed from the system by the initial (legacy) disturbance. The effects are expected to be multiplicative in the case of mixed perturbation **(D)**, as additional organisms are removed from the community by the second disturbance, resulting in a less diverse community that is less able to reorganize and recover following disturbance.

Compounded perturbation of soil may thus have opposite effects on the soil bacterial community depending on whether the disturbances are mixed or homogeneous, but this is unclear, as the effects of the two types of perturbation have not been compared for the same community, and the outcome of such perturbations may vary according to the soil matrix and its community. Consequently, in order to understand these effects, the two types of compounded perturbation should be compared for the same soil, under similar conditions. We hypothesized that these differing outcomes can be explained by mortality and the associated loss of microbial diversity, as well as the successional patterns that ensue and allow the colonization of the newly available niches (**Figure 1**). Systems with high species richness are expected to contain organisms with a broader array of environmental tolerance ranges, which should fare better across a wider range of environmental challenges or disturbances (Naeem and Li, 1997; Yachi and Loreau, 1999; Balser et al., 2001). In the case of homogeneous compounded perturbation, multiple similar disturbance events would have the strongest impacts on similar taxa and favor similar survivors, so the effect of the second event would be less perceptible (**Figure 1B**). In the case of mixed compounded perturbation, different taxa may be impacted by a subsequent different disturbance, resulting in a further erosion of the community’s diversity during recovery (**Figure 1D**).

Successional dynamics further obscure the impact of compounded perturbations on microbial communities. Following a first perturbation, tolerant and resistant organisms will be favored. As succession proceeds, however, these populations might be outcompeted by rapidly growing opportunists and eventually specialists, as easily digestible resources become scarce (Jurburg et al., 2017). Over time, resistant organisms are diluted out of the community by the arrival of new strategists (Placella et al., 2012), resulting in a community that is once again vulnerable to the disturbance (**Figure 1A**). Thus, as a community recovers from an initial perturbation, it will likely be initially more vulnerable to a novel perturbation (mixed compounded perturbation) and become more resistant the more time is allowed between disturbances, while the opposite pattern is expected in the case of homogeneous compounded perturbation.

Here, we explore the effect of disturbance legacy on the ability of the soil bacterial community to cope with a similar or a novel disturbance. To focus on the effect of the disturbances rather than environmental variability, we set up soil microcosms and exposed these to a model disturbance that consisted of an initial heat shock (along with unexposed microcosms), followed by a period of recovery (25 days), which allowed microbial communities to recolonize the soils in order to create a legacy. To verify the effect of compounded perturbations, these microcosms were then subjected again to model disturbances, which consisted of a similar heat shock heat-shock or a cold shock. Thus, a total of six treatments were applied: heat shock-heat shock (homogeneous compounded perturbation), heat shock-cold shock (mixed compounded perturbation), heat shock-control (single perturbation), control-heat-shock (single perturbation), control-cold shock (single perturbation), control–control (no perturbation). By frequently monitoring the bacterial community composition in the aftermath of these extreme selective sweeps, we evaluated whether the presence and the type of a disturbance legacy affect the successional dynamics of the soil bacterial communities.

## Materials and Methods

### Microcosms

A total of 205 microcosms were prepared by adding 50 g of fresh soil to 200 ml glass jars covered with loose aluminum foil caps. Microcosms were constructed using the top 15 cm of a loamy sand soil (soil-water pH 5.04) collected in April 2013 from a well-characterized agricultural field in Buinen, Netherlands (52°55′N, 6°49′E), where seasonal variations in biochemical parameters have been previously characterized (Pereira et al., 2011, 2012). Prior to the experiment, soils were homogenized by sieving through a 4-mm sieve and allowed to stabilize for 1 month at 4°C. After the preparation of the microcosms, soils allowed to stabilize for 2 weeks, under the same conditions as the experiment: microcosms were maintained at 21°C, partially shielded from light in a temperature-controlled greenhouse, and at 65% water-holding capacity (adjusted with sterile water). Sampling was done destructively in quintuplicate, at 10 sampling times (see below).

Microcosms were subjected to one of six treatments, which consisted of combinations of short-term model disturbances, applied in two phases. In the first phase of the experiment, half of the microcosms were subjected to an initial heat shock, followed by 25 days of recovery whereas the other half remained under control conditions for the same period of time. During the second phase, almost all microcosms (except controls) were subjected to additional disturbances, generating the following treatments: (1) an additional heat shock (heat–heat; homogeneous compounded perturbation), (2) a cold shock (heat–cold; mixed compounded perturbation), (3) no disturbance (heat–control; single perturbation), (4) a heat shock (control-heat; single perturbation), (5) a cold shock (control-cold; single perturbation), or (6) control (no perturbation), followed by an additional recovery phase that lasted 25 days. The 25-day interval between treatments was selected after initial microcosm experiments with identical soils and conditions revealed that bacterial communities were still recovering from a heat shock after 25 days (Jurburg et al., 2017). A detailed schematic of our experimental setup is provided in the Supplementary Figure S1. The duration of the heat shock was selected after recording the effects of increasing durations of microwave heating (15 s to 10 m) on the total copies of 16S rRNA transcripts, soil temperature, pH, and moisture loss, in order to generate a loss of between 33 and 57% of 16S rRNA transcripts (data available in Supplementary Table S2). During each heat shock, jars were uncovered, placed in an 800-watt microwave oven (R201ww Sharp, Utrecht, Netherlands), subjected to 90 s of heating at maximum intensity, adjusted for moisture loss, and covered immediately. The cold shock treatment consisted of placing jars in a -80°C freezer for 6 h. Soils were sampled 1 day prior to disturbance (T0) as well as on days 1, 4, 11, 18, and 25 days after disturbance (T1 to T25), at each phase of the experiment. Since we are interested in the effect of compounded perturbations, T0 to T25 refer to the second part of the experiment, whereas T-24, T-20, T-14, T-6, when mentioned, refer to the first phase.

### DNA and RNA Extraction

DNA was extracted from 0.5 g soil using the MoBio PowerSoil DNA Extraction Kit (MoBio Laboratories, Carlsbad, CA, United States) according to the manufacturer’s instructions, with three additional 30-s rounds of bead-beating (mini-bead beater, BioSpec Products, Bartlesville, OK, United States). The concentration and band size of the extracted products were checked by electrophoresis using a 0.8% agarose gel with a SmartLadder (Eurogentec, Liege, Belgium).

For the RNA extraction, 2 g of soil were placed in 5 mL of LifeGuard Soil Preservation Solution (MoBio laboratories, Carlsbad, CA, United States) for ∼24 h at 4°C, and then maintained in dry ice/-80°C until extraction, which took place 7 days after sampling. Extractions were performed with the RNA PowerSoil Total RNA Isolation Kit (MoBio Laboratories, Carlsbad, CA, United States) according to the manufacturer’s instructions. Extracts were re-suspended in 1 mM sodium citrate, quantified using a Quant-iT^TM^ RNA Assay Kit (range 5–100 ng; Invitrogen, Molecular Approaches, Eugene, OR, United States) on a Qubit^TM^ fluorometer (Invitrogen, by Life Technologies, Nærum Denmark). Samples with total RNA concentrations <20 ng μL^-1^ were discarded. Products underwent an optimized DNase treatment from the DNA-free^TM^ Kit (Ambion^®^, by Life Technologies,^TM^ Nærum, Denmark) protocol and were then subjected to reverse transcription using the Roche reverse transcription kit (Roche, Hvidovre, Denmark) with Random Hexamers (100 μM; TAG Copenhagen, Denmark). Further details are available in Supplementary S3.

### 16S rRNA Gene Copy Number and Transcript Quantification

Quantitative PCR of the 16S rRNA gene was run with reverse-transcribed RNA (cDNA) and DNA, respectively, using an ABI PRISM 7300 Cycler (Applied Biosystems, Darmstadt, Germany) targeting the 264-bp V5–V6 region using the primers 16SFP/16SRP (Bach et al., 2002). Reaction mixtures of 25 μL consisted of 12.5 μL SYBR Green PCR Master Mix (Applied Biosystems, Foster City, CA, United States), 0.5 μL of 20 mg mL^-1^ bovine serum albumin (Roche Diagnostics GmbH, Mannheim, Germany), 2 μL of forward and reverse primers (10 mM), and 1 μL of template cDNA or DNA at a concentration of 10 ng μL^-1^. Cycling conditions were as follows: 95°C for 10 min, followed by 39 cycles of denaturation at 95°C for 20 s, annealing at 62°C for 60 s, and extension at 72°C for 60 s; fluorescence was detected after annealing. The specificity of the products was confirmed by melting curve analysis and checked on a 1.5% agarose gel. A standard curve was generated using linearized plasmids containing a fragment of the 16S rRNA gene cloned from *Burkholderia* sp. spanning six orders of magnitude (10^2^–10^8^). Amplification efficiency (E) was calculated according to the equation, E = (10^-1/slope^ - 1). For all runs, 90% < E < 110%. The obtained data were log-transformed. The ratio of 16S rRNA transcripts to 16S rRNA gene copy number was used to estimate average ribosomes per cell.

### 16S rRNA Sequencing and Analyses

cDNA obtained from 10 ng of total RNA was used for 16S rRNA gene transcript amplicon sequencing, described in detail in Supplementary S3. Briefly, the primers 341F and 806R (Sigma–Aldrich, Brøndby, Denmark) flanking the V3 and V4 regions of the 16S rRNA gene were used to amplify a gene fragment of 460 bp (Yu et al., 2005; Berg et al., 2012). Sequencing of the 16S rRNA gene transcript amplicons was done using MiSeq reagent kit v2 (500cycles) and a MiSeq sequencer (Illumina Inc., San Diego, CA, United States).

Sequence analyses were prepared as follows: paired-end reads were mated and trimmed for primers using *Biopieces*^1^. Reads were quality-filtered with UPARSE (Edgar, 2013) with the following parameters: max expected error algorithm with –*maxee 0.5.* Dereplication was performed and singletons removed. OTUs were clustered at 97% using *usearch-cluster otus* and *usearch_global*. OTUs were chimera-checked with UCHIME against Greengenes 2011 (DeSantis et al., 2006). Representative reads picked by usearch were classified using Mothurs Wang implementation against the RDP trainset PDS v9 (Schloss et al., 2009). Classifications were accepted at a threshold of 80% confidence at each taxonomic level. Qiime wrappers for PyNAST (Caporaso et al., 2010a), FastTree (Price et al., 2009), and *filter_alignment.py* (Caporaso et al., 2010b) were used to construct a phylogenetic tree. Alignments were built against the 2011 version of Greengenes (DeSantis et al., 2006) and filtered using parameters *–allowed_gap_frac 0.999999* and *–threshold 3.0.* Abundances of amplicon sequences were used as a measure of the composition of the microbial community.

### Statistical Analyses

All analyses were performed in the R environment (R Core Team, 2014). Prior to analyses, amplicon data were rarefied to 1474 reads per sample, representing 5603 OTUs, using *rarefy_even_depth* from the *Phyloseq* package (seed.number = 266315). Samples lost during processing are detailed in Supplementary Table S6.

Rarefied data was used to calculate taxonomic richness, measured as the number of OTUs per sample, and evenness, measured as Pielou’s evenness index (*vegan* package, Oksanen et al., 2013). Significant differences between the control and all other treatments were compared for each time point using a two-tailed *t*-test (*p* < 0.05).

The rarefied data were also used to examine beta diversity through a Principal Coordinates Analysis (PCoA) of weighted Unifrac distances, using the Phyloseq package (McMurdie and Holmes, 2013). The difference between treatments was evaluated with a PERMANOVA with 999 permutations using *adonis* from the *vegan* package. The recovery of community composition through time was evaluated through a Principal Response Curve (Van den Brink and Ter Braak, 1999). Significant differences between control and all other treatments were not calculated due to uneven sampling depth resulting from samples lost during processing (Supplementary Table S6). In order to select OTUs that responded to treatments or changed over time, multiple SIMPER analyses (Clarke, 1993) were performed, comparing differences between early (T1–T4) and late (T11–T25) recovery within treatments, as well as within recovery stages and between treatments. 46 OTUs accounted for 50% of the dissimilarity observed in pair-wise comparisons between all the conditions. The relative abundances of samples from each replicate were averaged (centered and scaled), and these were clustered according to their temporal abundance patterns (*vegan* package, Euclidean distance, Ward’s clustering). The significance of observed clusters was confirmed using 999 permutations (*ade4* Dray and Dufour, 2007).

## Results

### Total Level of Normalized 16S rRNA Molecules and α-Diversity

**FIGURE 2:**
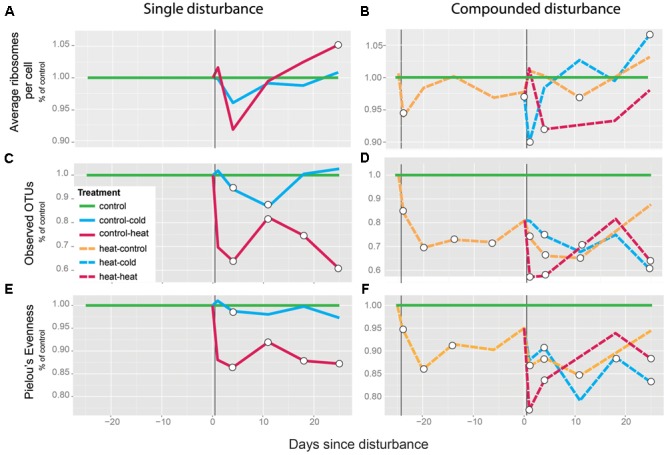
Effects of disturbance legacy of heat shock on the active community. Average ribosomes per cell **(A,B)**, richness **(C,D)**, and evenness **(E,F)** are shown as normalized ratios relative to the mean undisturbed control values for each respective time point. Average ribosomes per cell are measured as total 16S rRNA gene copies normalized by the number of 16S rRNA gene transcripts. Statistically significant differences between the undisturbed-control and each treatment along time are shown as hollow circles (two-tailed *t*-test, *p* < 0.05). Vertical black lines indicate the disturbance event. Normalizations of treatments with prior heat shocks relative to the heat–control treatment are available in Supplementary Figure S5. A list of the samples lost during extraction and processing is included in Supplementary Table S6.

In order to assess the effect of the single vs. the multiple disturbances on the bacterial communities in the soil, we first calculated the ratio of 16S rRNA molecules to gene copies (cDNA/DNA) by qPCR across treatments as a measurement of average ribosomes per cell and a proxy of growth rate (Campbell et al., 2011). In previously undisturbed soils, the heat shock (control-heat) resulted in a non-significant, 8.3% average decrease of normalized 16S rRNA gene transcripts compared to the controls on T4 (*p* = 0.68 for two-tailed *t*-test between treated and control). This was followed by a rapid return to pre-disturbance levels (**Figure 2A**). A significant decrease was observed for the soils from the heat–heat treatment, which exhibited an 8.1% average decrease compared to control soils on T4 (*p* = 0.02 for two-tailed *t*-test between treated and control). This was similarly followed by a rapid return to pre-disturbance levels (**Figure 2B**), reaching similar levels as the control soils afterward. In previously undisturbed soils, the cold shock (control-cold) had no significant effect on the ratio at any point during the experiment (*p* > 0.12 for all comparisons between control-cold and control treatments, **Figure 2B**). In contrast, in previously heat-treated soils, the cold shock (heat–cold) led to a 10% average decrease compared to undisturbed controls on T1 (*p* = 0.03 for two-tailed *t*-test between treated and control). This was followed by a rapid return to pre-disturbance levels (**Figure 2B**). Comparison of soils from the heat–heat and heat–cold treatments to soils from the heat–control treatment revealed similar patterns (Supplementary Figure S5A).

We also evaluated the effect of these treatments on α-diversity (total OTUs and Pielou’s J, **Figures 2C–F**). Regardless of prior disturbance, 4 days after disturbance (T4) the heat shock resulted in a significant decrease in OTU numbers (control-heat, average decrease 36%, *p* < 0.01; heat–control, average decrease 25%, *p* = 0.01; heat–heat, average decrease 42%, *p* < 0.01, **Figures 2C,D**) and evenness (control-heat, average decrease 13%, *p* < 0.01; heat–control, average decrease 11%, *p* = 0.02; heat–heat, average decrease 16%, *p* < 0.001, **Figures 2E,F**). The effects in soils with prior disturbance were more severe, however, as in T1 soils from the heat–heat treatment exhibited average reductions of 42% *p* = 0.001 and 23% *p* < 0.001, in richness and evenness, respectively. In contrast, soils from the control-heat treatment exhibited non-significant 30% decreases (*p* = 0.2) in richness and 12% decreases (*p* = 0.1) in evenness. The decrease in OTU richness and evenness remained significantly different from the control soils throughout the experiment, except for the treatment heat–heat at T18.

Relative to the heat shock, the cold shock was a minor disturbance. In undisturbed soils, the cold shock resulted in mildly significant 6% (*p* = 0.047) and 1% (*p* = 0.034) reductions in richness and evenness, respectively, relative to controls. While these soils recovered evenness levels comparable to controls by T11, richness exhibited a 13% (*p* = 0.030) decrease relative to controls at this time. In contrast, richness and evenness in the heat–cold treatment followed a similar recovery trajectory as in the heat–control treatment, but rather than recover, these parameters decreased over time, resulting in 40% (*p* = 0.001) and 20% decreases in richness and evenness relative to controls, respectively, on T25 (*p* = 0.009).

### Bacterial β-Diversity and Community Composition

**FIGURE 3:**
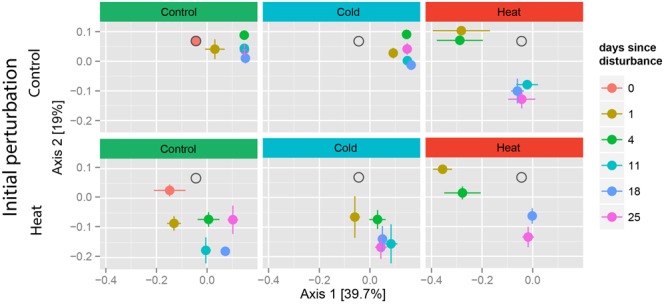
Recovery trajectories of community composition over time. Principle coordinates analysis (PCoA) of weighted Unifrac distances between samples. A single PCoA was separated according to disturbance type and soil history. Pre-disturbance controls (0 days) are only shown for undisturbed treatments. Gray hollow circles indicate the location of samples from the T0 controls, for reference. A list of the samples lost during extraction and processing is included in Supplementary Table S6.

Heat stress had a remarkable effect on community structure, regardless of prior stress, as shown by the displacement of samples along both axes of the PCoA (**Figure 3**). Cold stress had a similar effect on community composition only when applied in compounded perturbation (heat–cold), but not as single disturbance. A PERMANOVA of the weighted-Unifrac distances between samples showed a significant effect of treatment (*p* < 0.001), time since disturbance (*p* < 0.001) and the combination of these two factors (*p* < 0.001) on community composition (**Figure 3** and Supplementary Table S4). In the control-heat as well as the heat–heat treatments, samples exhibited temporal clustering. Notably, these shifts in community composition occurred in two stages: in the first stage, samples from T1 and T4 clustered together and were the most different from the undisturbed, control samples, while in the second stage samples from T11–T25 clustered together (**Figure 3**, right panels).

In contrast, soils exposed to the cold treatment did not change over time and clustered together with the undisturbed controls. Relative to the heat–heat and control-heat treatments, all samples from the control-cold treatment clustered closely together in the PCoA, suggesting only a slight effect of the cold treatment as well as low between-replicate variation. Similarly, samples from the heat–cold treatment clustered closely together in the PCoA, but were also statistically similar to each other (*p* > 0.08 for all comparisons), suggesting a higher between-replicate variability, possibly as a result of the past heat disturbance.

**FIGURE 4:**
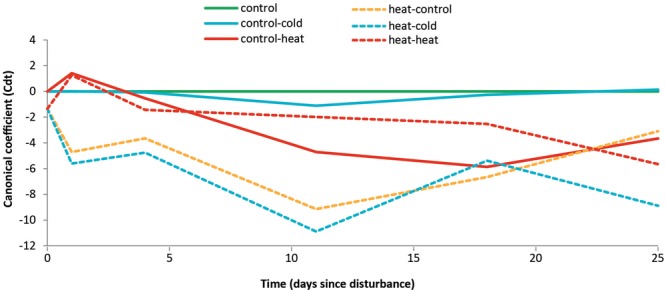
Effects of a prior heat shock on the recovery of the bacterial community composition. Principal response curve of OTU abundances over time. Line color represents different disturbance types (blue, red, and orange) or the undisturbed control soil (green). Canonical coefficients indicate the distance between the community composition of samples from all treatments relative to the undisturbed control. Previously undisturbed treatments are shown as solid lines, and previously disturbed treatments are shown as dashed lines. The variance explained by Time is 18.6%; the variance explained by Time^∗^Treatment is 39.6%.

We constructed a principal response curve (PRC, **Figure 4**) in order to compare the recovery trajectories of the bacterial communities exposed to the different treatments relative to the undisturbed control. For heat-shocked soils, the changes strongly depended on time since disturbance: on T4, the bacterial communities from the control-heat and heat–heat treatments exhibited compositions that were similar to each other, but differed from the other previously heat-shocked soils (heat–control and heat–cold treatments). The structure of the communities of soils from the heat–heat treatment was less affected than of those of soils from control–heat treatments, as the former increasingly diverged from the controls throughout the experiment. On the other hand, the communities in soils from the control–heat treatment exhibited a greater deviation from those of the control soils between T11 and T18; these showed signs of recovery by T25. However, comparing these two treatments to the heat–control revealed that communities undergoing heat stress did not recover, remaining different from the control, undisturbed soils until the end of the experiment. The lack of temporal variation in the heat–control samples (**Figure 3**) further suggests that they reached a stable alternative state. Furthermore, a strikingly different pattern was observed for the cold-shocked soils: soils from the control-cold treatment showed no effect of the cold shock, while soils from the heat–cold treatment exhibited the largest deviations in bacterial community composition of all treatments, relative to the controls. This deviation increased over time, showing no signs of short-term recovery in community structure.

### OTUs Explaining the Variation

**FIGURE 5:**
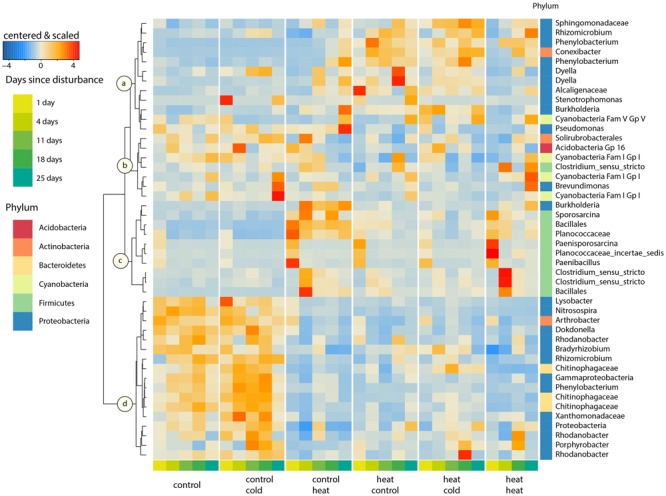
Core dynamic taxa. Heatmap of the Bray–Curtis distances of 46 OTUs, which explain 50% of the separation between treatments and between successional stages within each treatment. OTUs were selected according to pairwise SIMPER analyses and clustered using Ward’s method. These taxa represent 41.6% of the total community. Abundances were averaged per time point, and centered and scaled prior to plotting. The phylum membership of each OTU is displayed in the right column. OTUs at the lowest taxonomic classification level are listed on the right. Taxa were clustered into four groups (a–d) according to their temporal response patterns. A list of the samples lost during extraction and processing is included in Supplementary Table S6.

We selected the OTUs which explained 50% of the differences between the communities with respect to treatments and sampling time. These 46 OTUs clustered according to four response patterns, denoted as cluster *a, b, c*, and *d* (**Figure 5**). The OTUs in cluster *a* consisted of phyla that have been previously shown to exhibit delayed, positive responses to extreme environmental change (α-, β-, and γ-Proteobacteria, Placella et al., 2012). These taxa were present in low numbers in the controls and increased in relative abundance following the heat shock, gradually increasing during the second successional stage (T11–T25). Thus, this cluster did not seem to respond to heat directly, but rather to the potential release of nutrients caused by the death of heat-sensitive microbes. This second-phase increase in abundance occurred earlier in the heat–heat treatment than in the control-heat treatment. For example, the average relative abundance of a conspicuous OTU classified as a *Phenylobacterium* sp., gradually increased from 0.01% 1 day after heat shock to 3.6% on T25 of the control-heat treatment, but achieved a relative abundance of 5.12% by T4 of the heat–heat treatment. Moreover, the high abundance of this cluster in heat–control and heat–cold indicated that they successfully – and in a stable fashion – colonized soils after heat stress. Cluster *b* contained OTUs assigned to the Cyanobacteria, Firmicutes, Proteobacteria, Actinobacteria (one OTU) and Acidobacteria (one OTU), but showed no clear patterns. Cluster *c* contained taxa that were tolerant to, or favored by, the heat shock, and included members of the Firmicutes and one *Burkholderia* sp. The relative abundance of these taxa peaked during the first successional stage (T1–T4). Some taxa from cluster *c* remained at higher relative abundances throughout the rest of the experiment in soils without previous exposure to heat (control-heat), but their absence in soils pre-treated with heat (heat–control, heat–cold, and heat–heat) indicate that they were rapidly suppressed during soil colonization, possibly by OTUs from cluster *a*. For example, an OTU from cluster *c* assigned to spore-forming *Sporosarcina* increased in average relative abundance from 0.8% in controls to 5.7% 4 days after heat disturbance, and maintained this abundance on T18 for the control–heat treatment, but had decreased to 1.6% in the heat–heat treatment at this time. Several taxa exhibited pronounced peaks in relative abundance following the treatment in the control–heat treatment: an OTU assigned to the *Planococcaceae* increased in average relative abundance from 1.4% of the community in controls to 11.7 and 6.8% on T1 in the control–heat and heat–heat soils, respectively, and then decreased. Other taxa exhibited peaks in soils from the heat–heat treatment, but experienced rapid decreases thereafter regardless of prior heat shocks: an OTU assigned to *Paenisporosarcina* increased to 5.1 and 10% of the community in the control–heat and heat–heat treatments, respectively, but decreased to less than 1% of the community thereafter. Finally, cluster *d* contained only rare (less than 1% on average) members of the Proteobacteria (i.e., *Porphyrobacter* sp., *Rhodanobacter* sp.) and Bacteroidetes (three *Chitinophagaceae* OTUs), which were most abundant in soils which were not exposed to a heat shock (undisturbed control and control–cold treatments), but exhibited average relative abundances below 1% for all treatments. Interestingly, some taxa (a *Chitinophagaceae* OTU and *Bradyrhizobium*) exhibited mild increases in response to the control-cold and heat–cold treatments during T4-T18, suggesting the existence of subtle, consistent successional patterns in response to the cold shock.

## Discussion

Understanding how disturbances, or selective pressures shape communities and their response to further perturbation is fundamental to our knowledge of the dynamics of the soil biota over time. However, the overwhelming diversity and variability found in soil bacterial communities and the heterogeneity of the environment which surrounds them are common obstacles for the detection of clear, replicable patterns of community assembly. Here we made use of extreme, transient disturbances to evaluate community assembly during secondary succession. By focusing on simplified microcosms and model, highly controlled disturbances rather than natural environments and their natural variations, we were able to demonstrate that disturbances can trigger successional dynamics in soil microbiomes, which is analogous to secondary succession in macroecology (Placella et al., 2012). We also showed that these dynamics are much larger in response to compounded perturbations that are experienced in rapid succession. By monitoring the soil bacterial community after either an extreme heat shock or a cold shock and prior or not to a heat shock, we examined how such a compounded perturbation affects secondary successions, whether the identity of the legacy prior to perturbations (i.e., heat–heat vs. heat–cold) affects the outcomes and which bacterial types respond to each case.

The selection of extreme disturbance treatments in this experiment allowed us to clearly detect different successional dynamics depending on the combination of disturbances. Similarly, the highly controlled microcosm environment reduced the chance that other perturbations affected our community’s recovery trajectory. Thus, this experiment serves as a basis for modeling bacterial secondary succession by providing a rigorous study of bacterial community recovery from disturbance and is not meant to emulate natural conditions, in which dispersal is much greater, disturbances are more subtle, and other organisms (i.e., mesofauna and plants) may play a role in modulating the observed dynamics.

### Response of Soil Bacterial Communities to Single Disturbances

Throughout this study, we defined disturbance as an event that alters the soil environment and has possible repercussions for the local microbial community, or directly alters that community (Rykiel, 1985). Extant studies of the soil microbial community’s responses to disturbance vary in the choice of sampling times after disturbance, from a few hours to several years (Shade et al., 2012). In a similar setup as in this experiment, we have previously shown that the main secondary successional stages take place within the first 18 days after recovery for soil microbial communities (Jurburg et al., 2017). In the previous study, as in this one, we used a microwave heat shock as a selective sweep. This was meant to allow us to analyze community recovery from a selective sweep rather than to mimic the effects of heating in natural settings. Unlike moisture stress, heating for several hours in an oven or chloroform fumigations, microwave heating is rapid, has an abrupt end, and while it may alter the soil nutrients, it does not persist in the environment afterward. This allowed us to observe the community struggling to occupy the newly available niches in the absence of the disturbance’s selective pressure, and ensured that no taxa grew during the disturbance. The effects of microwave heat disturbance were previously studied in order to select an exposure, which caused considerable mortality within the community (33–57%, Supplementary Table S2).

Community dynamics resulting from the heat shock occurred in two clear stages. During the first stage (T1–T4), the relative abundance of several Firmicutes increased, particularly *Sporosarcina* and *Paenisporosarcina*. Many members of the Firmicutes can form heat-resistant endospores, and may be stimulated to germinate by elevated temperatures (Galperin, 2013). In particular several, species of *Sporosarcina* have been documented to tolerate temperatures of 80°C for over 10 min, and to produce abundant spores within 3–4 days (Pregerson, 1973). The second stage (T11–T25) was characterized by a gradual increase in the relative abundance of several α-, β-, and γ-Proteobacteria including *Rhizomicrobium, Burkholderia*, and *Dyella*, respectively. While extremely diverse, members of these classes have been shown to increase in the aftermath of wet-up in a delayed fashion relative to the rest of the community (Placella et al., 2012), and are more abundant in soils with high carbon availability (Fierer et al., 2007). This pattern is consistent with our previous findings (Jurburg et al., 2017), as well as with the successional niche hypothesis (Pacala and Rees, 1998), in which following disturbance, individuals which are tolerant to the disturbance are initially favored, but are eventually displaced as they are outcompeted for the newly available niche space. This pattern indicates that the heat shock was strong enough to trigger successional dynamics. It is important to note, however, that the increases observed in relative abundances may have also resulted from a decrease in other taxa. Relative to the heat shock, the cold shock was mild and its effects were less obvious. A significant reduction in total OTUs was observed for T4–T1, suggesting that some taxa were vulnerable to the cold shock, but it is likely that these taxa were rare, as this mortality did not alter the community’s composition or evenness.

In the heat shock but not in the cold shock treatment, the speed of recovery following the disturbance varied and was dependent on the metrics used to assess it: the number of potentially active bacteria returned to pre-disturbance levels by T25 in all soils, while the community composition in soils exposed to heat at any point remained different from controls and showed no indication of recovery. The notion that microbial community structure is more vulnerable to disturbance and recovers more slowly than function has been previously shown in a meta-analysis (Allison and Martiny, 2008). Our results further highlight the complexity of bacterial communities relative to their growth rate, and the need to assess soil microbial recovery with more complex metrics that account for successional patterns.

### Response of Soil Bacterial Communities to Compounded Perturbation

The idea that multiple simultaneous or frequent disturbances may override an ecosystem’s recovery mechanism (Paine et al., 1998) has serious implications for today’s biosphere. Ecosystems are exposed to extreme environmental conditions at an increasing frequency as a result of climate change and anthropogenic activities. For soil microbial communities, these include increasingly intense and frequent dry-rewetting cycles and increasingly common intensive agricultural practices, among others. While soil bacterial communities were previously thought to be immune to disturbance due to their rapid regeneration times and wide tolerance ranges (Finlay et al., 1997), we have observed that successional dynamics resulting from disturbance may affect the soil bacterial community for 25 days after the disturbance (Jurburg et al., 2017). Thus, for at least 25 days, the recovering soil microbial community may be more vulnerable to further perturbation. Multiple studies have observed that soil microbiomes may acclimate, at least partially, to disturbances if they are pre-exposed (Fierer et al., 2003; Bérard et al., 2012; Bouskill et al., 2013). We tested whether compounded perturbation had a multiplicative effect on bacterial community structure if the disturbance was the same as the previous one, or whether it was different.

The effects of the heat–heat treatment were remarkably similar to those of the control-heat treatment, but successional dynamics seemed to intensify. For example, in soils from the control-heat treatment, several Firmicutes persisted at higher relative abundances than in the controls for the rest of the experiment, but they were quickly depressed to near-control abundances in soils from the heat–heat treatment (i.e., *Sporosarcina, Bacillales OTU, Planococcaceae OTU*). This may have resulted from the increased abundance of opportunistic Proteobacteria in previously disturbed soils, which would have been able to more rapidly displace Firmicutes if they were already in higher abundances in the community. Alternatively, some slow-growing, oligotrophic Proteobacteria may have benefitted from the release of complex compounds resulting from the heat disturbance, a well-known artifact associated with autoclaving soils. For example, a lowly abundant (0.001%) *Phenylobacterium* sp. increased in relative abundance more rapidly in soils exposed to the heat–heat treatment than in the control-heat treatment. *Phenylobacterium* strains are able to degrade phenolic compounds (Reznicek et al., 2015; Jacquiod et al., 2017), which were likely released following the heat shock, and were probably more abundant after the second shock. Furthermore, Firmicutes OTUs which increased only transiently in both treatments (i.e., *Planococcaceae incertae sedis* and *Paenisporosarcina*) exhibited a higher relative abundance during this “peak” in soils from the heat–heat treatment. We suspect that this occurred because in soils which had been pre-exposed to heat, heat-tolerant Firmicutes had already been selected for and sporulated, which gave them an advantage during the second heat shock.

We further applied a second, contrasting, selective sweep. Our initial hypothesis was that mixed compounded perturbation would be multiplicative if the two disturbances selected against very different portions of the population, thus we selected a transient disturbance that could be rapid, severe, and would not overlap with the heat shock in its selection pattern: a -80°C cold shock. Surprisingly, the cold shock, which had weak effects on the community on its own, had a drastic effect on soils with a prior heat shock, suggesting a multiplicative effect of the two disturbances. One possible mechanism for this phenomenon is that the heat stress destroyed aggregates and changed water availability within the soil matrix, making bacteria more vulnerable to freezing. Samples from the mixed compounded perturbation treatment (heat–cold) exhibited a significantly lower number of normalized 16S rRNA copies 1 day after the cold shock than those from any other treatment, at any other time in the experiment. It is important to note that changes in 16S ratios may have also arisen from changes in community dominance patterns by individuals with more or less 16S rRNA gene copies (Nemergut et al., 2015; Nunes et al., 2016). Community structures in soils from this treatment increasingly deviated from all other treatments over time. The lowered evenness in the communities from the heat–cold treatment relative to those in the heat–control treatment suggests that the cold disturbance, in combination with the prior heat shock, disproportionately altered the dominance patterns. This aligns with an earlier finding that suggested that community evenness is crucial in favoring functional stability under stress in communities of denitrifying bacteria (Wittebolle et al., 2009). In our experiment, the decrease in evenness may have been facilitated by the high mortality and successional patterns triggered by the prior heat shock: the second disturbance roughly aligned with the second successional stage, in which rapid-growing opportunists dominate the nutrient-rich environment. In this experiment, these were predominantly Proteobacteria, which were not visibly affected by the cold shock. Thus, it is likely that in the recovering soil, which was already dominated by Proteobacteria, a second disturbance that removed a small portion of the community, further favored the opportunistic taxa. Furthermore, it is important to note that the effect of the initial heat shock was still visible in samples from the heat–control treatment, which maintained lower levels of α-diversity throughout the experiment, likely due to the permanent removal of members of the community that were not heat-resistant. We limited the influence of immigration in our experiment by partially covering the microcosms, so it is possible that this diversity would have been recovered through immigration over time in natural systems. These results indicate that samples from heat–cold and heat–heat treatments were still recovering at the time of the second disturbance.

Our results align with our initial conceptual framework (**Figure 1D**) and shed light on the intricacies of compounded perturbation. Clearly, the chronology and type of disturbance events in soil are important in determining the outcome of additional perturbations. Had the weaker cold shock taken place before the heat shock, perhaps the effects would have been weaker, however, further work is needed to determine how the order of disturbances affects microbial communities. Furthermore, it is likely that the timing of the second disturbance played a crucial role in determining the effects we observed. Had the second cold and heat shocks taken place 4 or 40 days after the first heat shock, the exposed community—and its ability to recovery—would have been very different. Indeed, Kim et al. (2013) have shown that increasing the frequency of disturbance can have catastrophic results on the community: soil bacterial communities subjected to an increasing frequency (every 7, 14, 28, and 56 days) of dilution into sterile soil collapsed when the dilutions were weekly, resulting in highly ‘erratic’ community compositions (Kim et al., 2013). As in our experimental setup, their disturbance (90% dilution) was designed to evaluate community assembly during secondary succession rather than the implications for natural environments. In another experiment, Ho et al. (2016a) showed that the susceptibility of *Methylococcus*/*Methylocaldum*-related methanotrophs depended on the frequency of dry-rewetting cycles in rice field mesocosms, and that different groups of methanotrophs exhibited specific responses to dry-rewetting cycles, their frequency, or the cumulative effect of both (Ho et al., 2016a). While we did not observe any indications of community collapse, it is possible that a higher frequency of perturbation would have had a similar effect on our experimental communities. Further research is necessary to quantify the multiplicative effects of mixed compounded perturbation on microbial community recovery.

Previous theoretical work has concluded that a soil microbial community’s resilience is largely determined by the soil’s exposure to disturbances in the past (Hawkes and Keitt, 2015). Our findings are consistent with this notion, and shed light on the intricacies of soil microbial succession with regards to legacy effects. For example, traditional successional theory, which has been developed based on the study of larger organisms (i.e., plants and animals) posits that in successional gradients driven primarily by competition, an increased biodiversity and the resulting competitive pressure may slow down the successional dynamics (Drury and Nisbet, 1973). In our system, the lowered community diversity in soils pre-exposed to a heat shock relative to that in the undisturbed soil may have resulted in lowered numbers of competitors for the resources available after the heat shock, and thus a faster transition toward the opportunists that are characteristic of the second successional stage. In this way, soils with a disturbance legacy have become ‘specialized’ in recovering from a specific perturbation. We did not study whether this affects various community functions such as nitrification or respiration, but this warrants further research. Our results from the mixed compounded perturbation treatment show that this specialization comes at a cost, however. Soils that had been pre-exposed to a heat disturbance exhibited disproportionately larger shifts in community composition in response to a weaker (cold) disturbance than soils without this prior heat shock.

### Implications

Our study reveals the complexities of soil microbial community recovery from disturbance, and highlights the importance of considering a community’s history when evaluating its resilience. It is conceivable that the disturbances’ multiplicative effects arose from the restructuring of the soil matrix. Alternatively, they could arise from the erosion of community diversity and the alteration of ecological relationships within the community. This experiment reveals that in soil microbial communities, compounded perturbation accelerates or fundamentally alters successional patterns, depending on the identity of the legacy disturbance. The model disturbances used here were selected because they had not been experienced by the soils in the past; however, further research is necessary to determine to what extent these results hold true if the soils have been regularly exposed to the perturbation in the past (i.e., tilling, soil contamination by heavy metals, etc.), and to understand the role of abiotic factors such as soil type, in buffering the microbiota from environmental changes. For example, a recent experiment showed that the response of the methanotrophic communities in rice paddy soils to heat and desiccation stress is independent of prior exposure to the stressor (Ho et al., 2016b). The detection of successional stages in recovery emphasize the need to design experiments with sufficient temporal resolution in order to distinguish whether the sample comes from a recovered community, or from one undergoing succession. From a management perspective, our results highlight the potential vulnerability of soils exposed to multiple, different environmental pressures, and the need to consider time since disturbance in soil management regimes. This is particularly important in a world in which environmental fluctuations are expected to intensify, and soil microbial communities will need to be able to withstand a wide range of fluctuations in order to maintain their ecological integrity.

## Availability of Data and Material

The dataset supporting the conclusions of this article are publicly available NCBI’s Sequence Read Archive, under project ID PRJNA329541, https://www.ncbi.nlm.nih.gov/bioproject/PRJNA329541.

## Author Contributions

SDJ and JFS designed the study. SDJ performed the microcosm experiment, statistically analyzed data and wrote the first draft of the manuscript. IN performed RNA-related laboratory work. AB performed sequence analyses. JFS, SJS, and AP provided materials. JDVE, SJS, SJ, and JFS actively participated in data analysis, interpretation and writing. All authors contributed substantially to revisions.

## Conflict of Interest Statement

The authors declare that the research was conducted in the absence of any commercial or financial relationships that could be construed as a potential conflict of interest.

## Footnotes

1 www.biopieces.org
