# Supplementary material for: Legacy Effects on the Recovery of Soil Bacterial Communities from Extreme Temperature Perturbation
Source: Front Microbiol. 2017 Sep 25;8:1832. doi: 10.3389/fmicb.2017.01832 (PMC5622210; doi:10.3389/fmicb.2017.01832)
Supplement: Supplementary file 1 [file Data_Sheet_1.DOCX]

***Supplementary Material***

**Legacy effects on the recovery of soil microbial communities from perturbation**

Stephanie D. Jurburg^*^, Inês Nunes, Asker Brejnrod, Samuel Jacquiod, Anders Priemé, Søren J. Sørensen, Jan Dirk Van Elsas, Joana F. Salles.

***Correspondence:**

Stephanie D. Jurburg

[s.d.jurburg@gmail.com](mailto:s.d.jurburg@rug.nl)


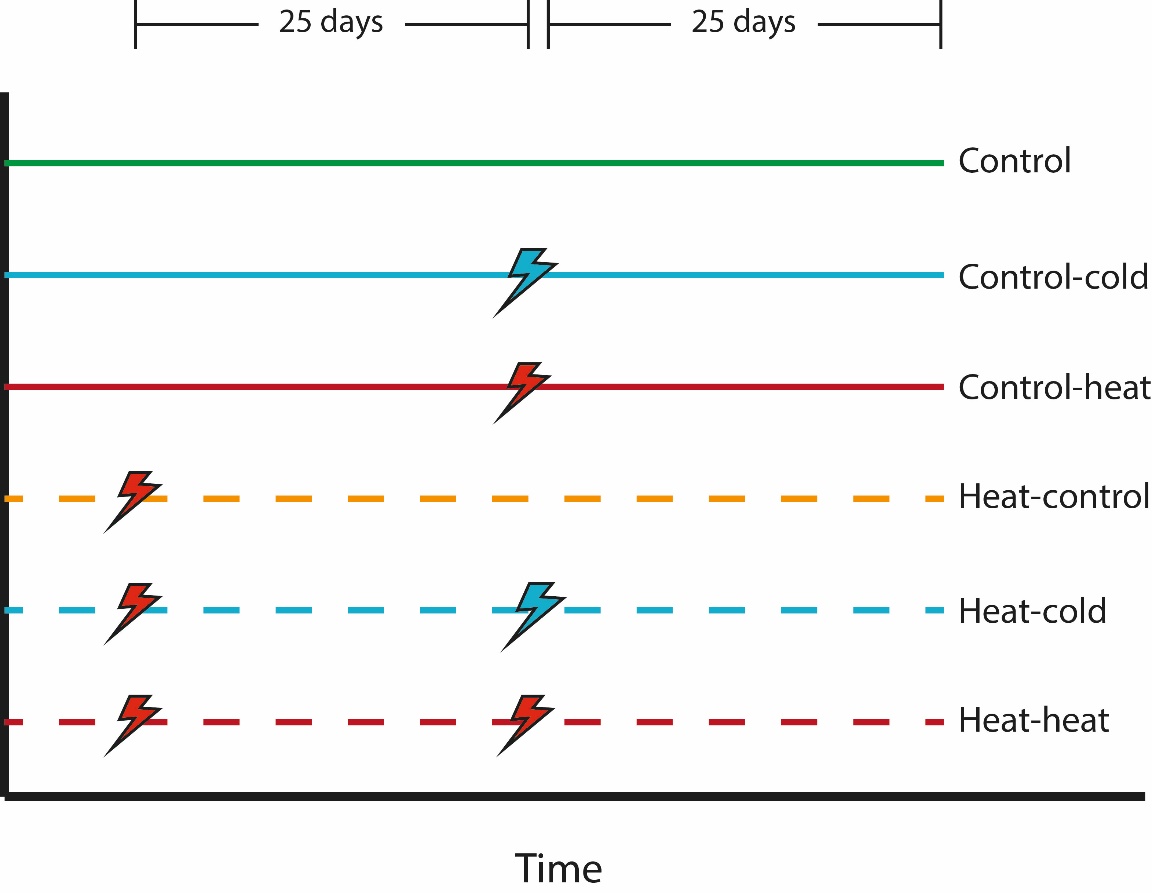


Figure S1. **Schematic description of the experimental setup**. Lightning symbols denote a heat shock (red) or a cold shock (blue) disturbance. Six treatments were developed by applying combinations of heat shocks, cold shocks, or control conditions, and allowing 25 days of recovery between disturbances.

| **Duration** | **log gene copies of rRNA transcript p/gram dry soil** | **Temperature** | **pH** | **Water Loss**  **(g/g dry soil)** |
| --- | --- | --- | --- | --- |
| Control | 9.9 | 22 | 4.99 | 0.00 |
| Control | 10.7 | 21 | 4.97 | 0.00 |
| Control | 10.3 | 21 | 4.98 | 0.00 |
| Control | **9.57** | 21 | 4.96 | 0.00 |
| 15 Seconds | 10.5 | 27.5 | 4.95 | 0.01 |
| 15 Seconds | 10.5 | 30 | 5 | 0.01 |
| 15 Seconds | 9.72 | 27 | 4.98 | 0.01 |
| 15 Seconds | 10.8 | 26 | 4.97 | 0.04 |
| 30 Seconds | 10.2 | 39 | 4.99 | 0.02 |
| 30 Seconds | 10.3 | 42 | 5 | 0.01 |
| 30 Seconds | 10.2 | 34 | 4.5 | 0.01 |
| 30 Seconds | 10.2 | 30 | 5.01 | 0.01 |
| 1 Minute | 10.2 | 55.5 | 4.97 | 0.02 |
| 1 Minute | 10.1 | 52 | 4.98 | 0.02 |
| 1 Minute | 10.1 | 54 | 5.01 | 0.01 |
| 1 Minute | 10.1 | 48 | 4.99 | 0.02 |
| 2 Minutes | 9.84 | 72 | 4.95 | 0.02 |
| 2 Minutes | 9.92 | 71 | 5 | 0.03 |
| 2 Minutes | 9.86 | 55 | 4.96 | 0.03 |
| 2 Minutes | 10.1 | 66 | 4.93 | 0.03 |
| 5 Minutes | 9.28 | 72 | 4.95 | 0.12 |
| 5 Minutes | 9.3 | 70 | 4.96 | 0.15 |
| 5 Minutes | -- | 65 | 5.02 | 0.13 |
| 5 Minutes | -- | 55 | 4.88 | 0.15 |
| 10 Minutes | -- | 82 | 5.02 | 0.23 |
| 10 Minutes | -- | 87 | 5.03 | 0.23 |
| 10 Minutes | -- | 86 | 5.03 | 0.23 |
| 10 Minutes | -- | 85.5 | 5.02 | 0.24 |

Table S2. **Effect of increasing exposure to microwave radiation on the soil microcosms.** No RNA was extractable at the highest exposures. The 16S rRNA transcript counts from one control sample (bold, underlined) was excluded from calculations.

S3**. Supplementary material and methods**

**RNA processing**

200 ng of total RNA were treated using 2 µL of 10X Dnase I buffer and 1 µL of DNase I. Incubation was performed for 1h at 37°C with mixing at each 10 minutes. 5 µL of DNase inactivation reagent were then added and incubation at room temperature for 5 minutes with frequent mixing for precipitate re-suspension was made. Supernatant was recovered after centrifugation at 10000 g.

cDNA was obtained using the Roche reverse transcription kit (Roche, Hvidovre, Denmark) with Random Hexameres (100 µM; TAG Copenhagen, Denmark). Reaction mixes were constituted by 4 µL of 5x Strand buffer, 2 µL of DTT (0.1 M), 1 µL of dNTP (10 mM), 1 µL of Random Hexameres, 10 ng of template DNase treated RNA and water to a total volume of 20 µL. Mixes were incubated in a Peltier Thermal Cycler (DNA Engine DYAD™, USA) in a one cycle protocol as following: 2 minutes at 42°C followed by the addition of 1 µL of reverse transcriptase (50 U/µL; Roche, Hvidovre, Denmark), 40 minutes at 42°C, 30 minutes at 50°C and 15 minutes at 72°C to stop the reaction.

**16S rRNA transcript sequencing**

The primers 341F (5’CCTACGGGRBGCASCAG-3’) and 806R (5’GGACTACNNGGGTATCTAAT-3’) (Sigma-Aldrich, Brøndby, Denmark) flanking the V3 and V4 regions of the 16S rRNA gene were used to amplify a gene fragment of 460 bp. The chosen primer set have previously been successfully used to target bacteria and archaea but with a higher matching efficiency for bacteria (Yu *at al.*, 2005; Berg *et al.*, 2012). The PCR mixes were constituted by 2.0 µL of 10X AccuPrime™ PCR Buffer I containing 15 mM of MgCl2 (Life Technologies, Nærum, Denmark), 0.12 µL AccuPrime™ Taq DNA Polymerase (2 units/µl, Life Technologies, Nærum, Denmark), 1 µL of each primer (10 µM), 1 µL of a 1:10 dilution of cDNA as template and water to a total volume of 20 µL. The first PCR reactions were performed in a Peltier Thermal Cycler (DNA Engine DYAD™, USA) according to the following conditions: an initial activation of the hotstart polymerase at 94°C for 2 min, followed by 35 cycles of denaturation at 94°C for 20 s, annealing at 56°C for 20s and extension at 68°C for 30s, with a final extension at 68°C for 5 min. Concentration of amplified PCR products was measured by Pico Green (Life Technologies, Nærum, Denmark) using a LightCycler 96 (Roche, Hvidovre, Denmark). Addition of adapters and indexes to DNA fragments was done in a second PCR where mixes were composed by: 2.0 µL of 10X AccuPrime™ PCR Buffer II containing 15 mM of MgCl2 (Invitrogen, Tåstrup, Denmark), 0.12 µL of AccuPrime™ Taq DNA Polymerase (2 units/µl, Life Technologies, Nærum, Denmark), 1.0 µL of each fusion primers (MS_515f_F IndexNo and MS_806r_R IndexNo; 10 µM), 2 µL of PCR product obtained in the previous PCR and water to a total of 20 µL. A Peltier Thermal Cycler (DNA Engine DYAD™, USA) was used to perform the second PCR reactions in the following way: initial activation of the hotstart polymerase at 94°C for 2 min, followed by 15 cycles of denaturation at 94°C for 20 s, annealing at 56°C for 20s and extension at 68°C for 30s, with final extension at 68°C for 5 min. The amplified PCR products were incubated at 70°C for 3 min and then placed on ice. Obtained fragment has approximately 400 bp including tag primers. Purification of PCR products was obtained using the AmPure XP Bead Purification Kit (Beckman Coulter, Copenhagen, Denmark) accordingly to the manufacturer instructions and applying 15 µL of AgencourtAMPure XP per reaction (0.75X of the total volume). The concentration of the purified second PCR products was measured by Pico Green (Life Technologies, Nærum, Denmark) in a LightCycler 96 (Roche, Hvidovre, Denmark) and equal amounts of DNA were pooled in a 1.5 mL tube. The pooled sample was concentrated using DNA clean and concentrator-5 kit (Zymo Research, Irvine, CA, USA).

Table S4. PERMANOVA using the effect of treatment, time since disturbance, and treatment * time since disturbance as factors to explain the compositional variance of the bacterial community (10000 permutations; weighted Unifrac dissimilarity matrix).

|  | **Df** | **MS** | **Pseudo-F** | **R2** | **P-Value** |
| --- | --- | --- | --- | --- | --- |
| **Time** | 10 | 0.28068 | 98.747 | 0.24291 | 0.001 |
| **Treatment** | 5 | 0.61147 | 215.122 | 0.26459 | 0.001 |
| **Treatment * Time** | 24 | 0.06775 | 23.837 | 0.14073 | 0.001 |
| **Residuals** | 143 | 0.02842 | 0.35177 |  |  |
| **Total** | 182 | 100.000 |  |  |  |


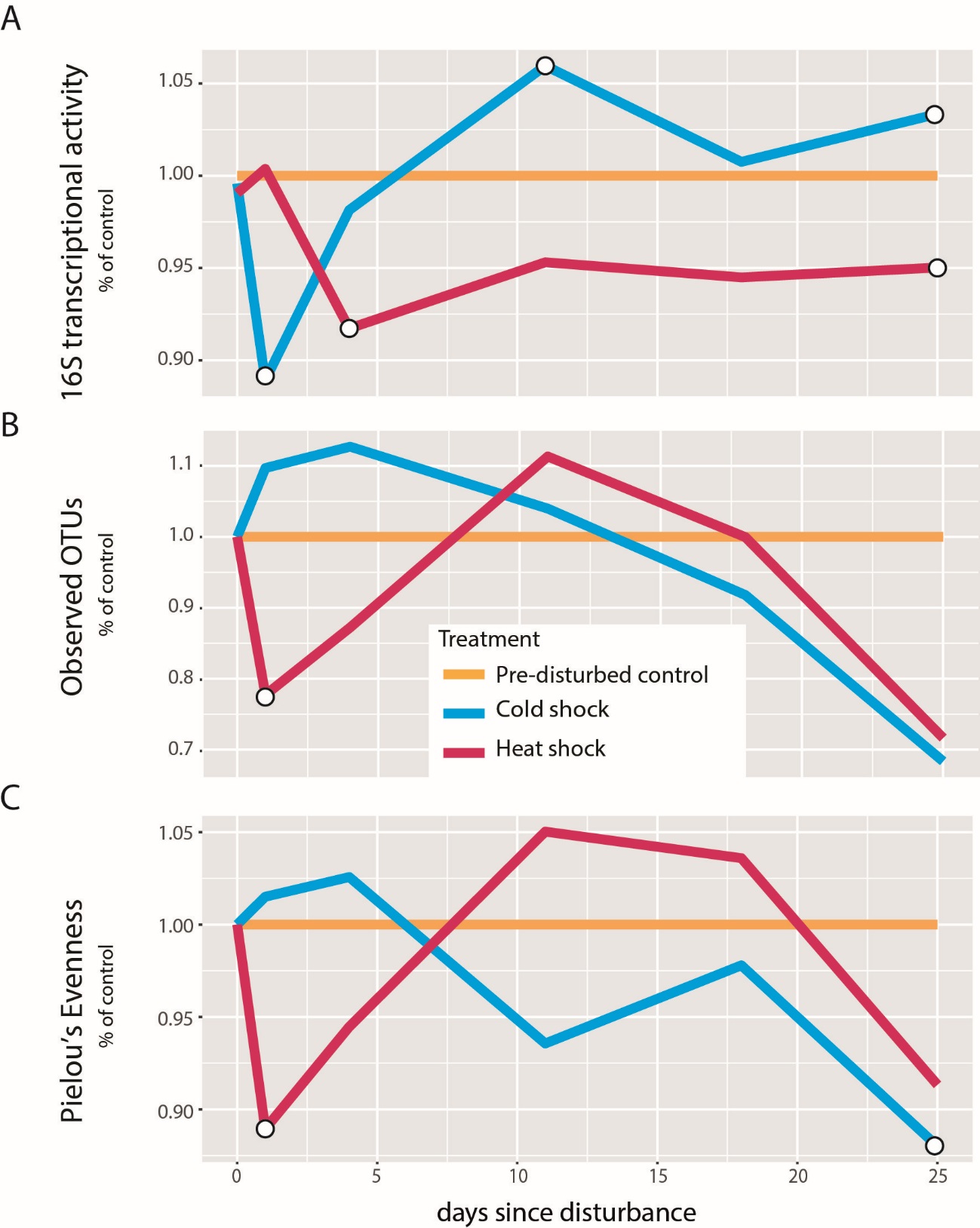


Figure S5. **Effects of disturbance legacy of a heat shock on the bacterial community.** 16S rRNA transcript numbers over genes (A), richness (B), and evenness (C) are shown as normalized ratios relative to the mean pre-disturbed control (heat-control) values for each respective time point. Statistically significant differences between the undisturbed-control and each treatment along time are shown as hollow circles (t-test, *p* < 0.05).

Table S6. **Summary of samples lost during extraction and processing**. See.xls file.
